# The impact of quality of information on classroom engagement with GenAI tools: integrating TAM and SOR framework

**DOI:** 10.3389/fpsyg.2026.1756708

**Published:** 2026-02-20

**Authors:** Fan Li, Juying Chen, Tongtong Shao, Jian Sun

**Affiliations:** 1 School of Music and Dance, Xihua University, Chengdu, Sichuan, China; 2 School of Foreign Languages, Weifang University of Science and Technology, Shouguang, Weifang, Shandong, China; 3 Faculty of Humanities and Social Sciences, City University of Macau, Taipa, Macau SAR, China

**Keywords:** quality of information (QI), qdoubao chatbot technology acceptance model (TAM), student engagement, perceived ease of use (PEU), perceived usefulness (PU)

## Abstract

This study investigates the role of quality of information (QI) in shaping students’ engagement with GenAI, specifically focusing on the Doubao chatbot in undergraduate English learning. Integrating the Technology Acceptance Model (TAM) with the Stimulus-Organism-Response (SOR) framework, the research examines how QI affects students’ perceived ease of use (PEU), perceived usefulness (PU), and ultimately their behavioral (BE), cognitive (CE), and emotional engagement (EE) with the tool. Data were collected from 110 undergraduate students. The findings revealed that QI significantly influences both PEU and PU, which subsequently affect students’ engagement. Notably, PEU exerted a stronger impact on engagement than PU. While PU drives behavioral engagement, it does not directly influence cognitive or emotional engagement. The results underscore the importance of high-quality, easy-to-use GenAI tools in enhancing student engagement. Developers and educators are encouraged to prioritize content quality and usability to foster deeper intrinsic motivation and long-term engagement.

## 1 Introduction

Educational technology has become a research hotspot in language education, transforming traditional teaching models through intelligent tools (Feng et al., 2025; Popenici and Kerr, 2017). Since 2022, the emergence of generative AI (GenAI) systems has reshaped information-seeking behaviors (Zhou and Li, 2024) and introduced novel solutions for foreign language learning (Badr et al., 2024). GenAI tools exemplify this trend and show strong potential for enhancing students’ learning experiences (Giannakos et al., 2025; Dahri et al., 2024). Powered by deep learning, these models can understand and generate human-like text, thereby providing personalized feedback and guidance (Lund and Wang, 2023; Lin et al., 2025; Iqbal et al., 2025). By enabling conversational practice and instant feedback, systems like Doubao help learners adopt new meaning-making practices and enjoy more personalized, creative, and productive learning experiences. They also increase exposure to the target language by simulating authentic, real-life communication scenarios. A rigorous understanding of these dynamics is essential for educators exploring and implementing transformative AI technologies in teaching (Liu and Ma, 2024).

While research on educational chatbots has accelerated, most studies emphasize benefits (e.g., satisfaction, efficiency) and experiential outcomes (Wu et al., 2020; Kim et al., 2019), and only recently have scholars begun to investigate different user responses to GAI chatbots (Ko and Lee (2024); Lee et al., 2025). This raises a fundamental question: what factors drive these divergent responses and, ultimately, students’ engagement? In particular, quality of information (QI) delivered by GAI chatbots appears central to user experience. Prior studies demonstrate that information quality strongly predicts user satisfaction and perceived usefulness, thereby shaping information processing and adoption (Erkan and Evans, 2016). Yet despite rapid uptake of GAI platforms in higher education, a notable research gap remains around student engagement (Maduku et al., 2024).

The Technology Acceptance Model (TAM) (Davis, 1989) offers a useful lens for explaining students’ engagement with educational technologies. Its two core constructs—perceived usefulness (PU) and perceived ease of use (PEU)—are established predictors of adoption (Venkatesh and Davis, 2000; Ifinedo, 2011; Tsai et al., 2021). However, TAM alone does not fully capture the multifaceted nature of contemporary AI systems; Davis (1989) therefore recommended incorporating external antecedents, and subsequent research has extended TAM with contextual factors across domains (Choi et al., 2023).

To address these gaps, this study develops an integrated framework that synthesizes TAM within the Stimulus–Organism–Response (SOR) paradigm (Mehrabian and Russell, 1974). In our model, the stimulus is the chatbot’s QI. It is composed of five dimensions: richness, relevance, accuracy, timeliness, and format. The organism comprises learners’ cognitive states—PU, PEU, and the response is students’ engagement. We argue that fine-grained QI dimensions differentially influence TAM appraisals, which in turn shape engagement with AI chatbots for language learning. To our knowledge, no prior study systematically examines how these QI dimensions affect TAM under the SOR framework within English learning context Al-Hoorie et al., 2025. This work therefore offers (1) a theory-driven integration of TAM, in which QI serves as a stimulus, (2) a disaggregated test of QI’s five dimensions and (3) empirical evidence with implications for the design of intelligent instruction systems in higher education—guiding developers to optimize interaction design to better meet students’ personalized needs.

## 2 Literature review

### 2.1 Quality of information (QI)

Recent work has begun to specify the QI for AI chatbots in education. Mun and Hwang (2025) identified five attributes of ChatGPT’s information quality—accuracy, richness, timeliness, format, and relevance—while Fu et al. (2024) proposed seven factors, including completeness, precision, timeliness, convenience, format, accuracy, and reliability. Across AI-supported contexts, QI consistently emerges as a pivotal determinant of trust, satisfaction, and continued use (Kumar and Daniel, 2016; DeLone and McLean, 2003). QI improves perceived ease of use. QI shapes whether users can obtain effective and reliable information that meets their needs (Ashfaq et al., 2020).

Within educational settings, TAM-based studies repeatedly found that higher QI predicts stronger PU of AI tools (Liu and Ma, 2024). For instance, Almaiah et al. (2016) showed that QI enhances both PU and PEU—two central determinants of technology acceptance. Nevertheless, important gaps remain. Most frameworks still reflect traditional information systems and are not fully adapted to the dynamic, conversational nature of GAI (Naidoo, 2023; Li et al., 2024). Real-time user evaluations are often underexamined, and few studies model how disaggregated QI dimensions affect learner engagement through an integrated pathway that includes PU, PEU. Addressing this gap would clarify how high-quality information fosters a more engaging and effective GAI-mediated learning environment, with benefits for both theory and practice.

Despite growing interest, the current literature have not fully integrated TAM into GenAI contexts. A comprehensive framework that integrates QI, TAM (PU/PEU), and engagement is therefore needed to explain learner engagement in GAI environments.

### 2.2 Technology acceptance model (TAM)

TAM was introduced by Davis (1989) and has become a cornerstone for predicting attitudes and acceptance of information technologies (Börekci and Çelik, 2024; Marangunić and Granić, 2015). It focuses on PU and PEU, which together account for intentions to adopt and continue using technologies King and He, 2006. PU emphasizes the practical value of technology and relevance to individual work responsibilities, while PEU focuses on minimizing barriers to entry and facilitating integration to workflow (Huang and Mizumoto, 2024). PEU is inherently context-dependent—users may perceive “ease” differently across tasks and settings (Lu and Gustafson, 1994). TAM has been widely applied to mobile and social technologies (e.g., Park et al., 2014) and adapted to GenAI contexts to examine how QI attributes shape PU, trust, and continuance (Mun and Hwang, 2025; Fu et al., 2024). Besides, TAM can be employed as a useful theoretical base to predict and understand users’ intentions to use e-learning (Al-Adwan et al., 2013).

According to the review analysis conducted by Salloum et al. (2019), QI was one of the most common external factors of TAM Lei and Wu, 2007. Their following empirical study involving 435 students in the United Arab of Emirates further verified the positive influence of QI on either PU or PEU of e-learning system (Salloum et al., 2019). Additionally, the research of Börekci and Çelik (2024) suggested the importance of PU and PEU in influencing Turkish university students’ behavioral intentions about AI. Building on TAM, some studies integrated Self-Determination Theory (SDT) to explain how autonomy, competence, and relatedness interacted with PU/PEU to drive intention (Nikou and Economides, 2017). Recent evidence indicated that autonomy and competence could reinforce the effects of PU and PEU on adoption in ChatGPT-based learning (Al-Mamary and Abubakar, 2025). Meanwhile, Huang and Mizumoto (2024) examined the relationship between L2 Motivational Self System (L2MSS) and TAM and revealed the notable correlation between them and the positive effect of ought-to L2 self on actual usage of ChatGPT. Yet, as Strzelecki (2024) notes, our understanding of students’ acceptance and actual use of GenAI remains incomplete. Therefore, this study aims to combine SOR and TAM to systematically investigate the influence of GAI QI on learners’ perception and engagement.

### 2.3 Engagement

Engagement refers to the degree of attention, involvement, motivation, and emotional investment that humans demonstrate when interacting with or performing tasks mediated by artificial intelligence (AI) systems Zhou et al., 2020. Engagement, in this sense, encompasses cognitive, emotional, and behavioral aspects, which collectively shape how users interact with GAI-mediated tasks (García-Navarro et al., 2024).

The concept of engagement itself has evolved significantly. Astin (1999) initially defined engagement as the physical and psychological energy students devote to their academic experience (Harter, 1981). This definition emphasizes commitment. Hu and Kuh (2002) expanded this definition, highlighting engagement as the effort students invest in activities that contribute to learning outcomes. Trowler (2010) framed engagement as a dynamic interaction, involving not only student effort but also institutional resources and practices.

Fredricks et al. (2004) offered a comprehensive framework, identifying three key dimensions of engagement: behavioral, emotional, and cognitive. Behavioral engagement captures observable actions like participation and attendance. Emotional engagement relates to students’ feelings about learning, such as interest and a sense of belonging Furrer and Skinner, 2003. Cognitive engagement reflects the mental effort students put into understanding complex concepts Chi, 2018 and mastering challenging skills Reeve and Tseng, 2011. Importantly, engagement exists along a continuum, where disengagement or withdrawal may occur in the absence of positive engagement. It is possible for students to show positive behavioral engagement (e.g., attending classes) while displaying negative cognitive engagement (e.g., rejecting the educational content).

### 2.4 SOR framework

The SOR framework (Mehrabian and Russell, 1974) has been widely adopted to explain how environmental inputs shape internal states and, in turn, behavioral outcomes Papaioannou et al., 2004. In educational contexts, particularly technology-rich, AI-enhanced settings, the model clarifies how features of the learning environment and tools influence learners’ cognitive and affective processes and subsequent behaviors such as engagement and satisfaction (Zhai et al., 2023; Pan et al., 2024).

Within SOR, stimuli comprise external factors impinging on the learner, including environmental, technological, and instructional characteristics (Chen et al., 2022). The organism denotes the internal mediating system, that is the cognitive appraisals and emotional states that interprets these stimuli (Jacoby, 2002; Mehrabian and Russell, 1974). Responses are observable outcomes such as engagement, satisfaction, or approach–avoidance intentions (Chen et al., 2022). This architecture is well suited to AI-mediated learning, where complex mental processes, including trust, perceived transparency, and affect are prominent (Jacoby, 2002; Mohamed Sadom et al., 2024).

Empirical studies in education and adjacent AI contexts consistently investigated the SOR chain. In online learning, learning experiences and technology quality as stimuli shape perceived learning effectiveness as an organismic state, which in turn elevates satisfaction and engagement (Cai et al., 2024). Social supports also function as salient stimuli: teacher and peer support increase perseverance and enjoyment, culminating in stronger work/learning engagement (Fan et al., 2024). Focusing on adoption mechanisms, educational technology characteristics influence trust and satisfaction, which mediate willingness to adopt and engage (Safdar et al., 2025). In organizational AI decision-making, perceived transparency and AI effectiveness trigger cognitive and emotional responses that shape trust and collaboration intentions, illustrating SOR’s portability to AI-rich settings (Cai et al., 2024). Collectively, this evidence supports the model’s explanatory power across diverse learning environments.

### 2.5 TAM and SOR in the Chinese context

Some studies have been conducted about TAM in the Chinese learning context, while the research on SOR remains relatively limited, not to mention the scarcity of studies investigating the integration of TAM and SOR. For instance, the research of Zhou et al. (2022) extended TAM to probe into factors affecting learners’ intention of using the online education platform, which demonstrated the effective role of external factors such as online course design, perceived system quality, and perceived enjoyment. Meanwhile, in accordance with TAM, PU and PEU were positively related to online learning effectiveness among emerging adult learners in China (Wu et al., 2023). To explore college students’ willingness to learn basketball through metaverse technology, Ren et al. (2022) included flowing variables based on TAM and revealed that flow experience, PU, and PEU could predict behavioral intentions. Moreover, PU and flow experience exerted an important effect on attitude (Ren et al., 2022).

Additionally, Yan et al. (2023) combined TAM and Theory of Planned Behavior (TPB) to examine Guangdong and Macau college students’ intention to participate in gamified online interactive platforms, verifying the mediating role of PU, perceived enjoyment, and perceived behavioral control between visual learning style and engagement intention. Likewise, Chen and Zhao (2022) integrated Self-Determination Theory (SDT) and TAM to understand Chinese EFL learners’ acceptance of gamified vocabulary learning apps. On one hand, autonomous motivation had the positive impact on both PU and PEU, whereas controlled motivation just positively affected PU. On the other hand, PU and PEU could influence learners’ behavioral intention and actual behavior (Chen and Zhao, 2022). Under the extended framework of expectation confirmation theory (ECT) and TAM, Ye et al. (2023) found the positive role of expectancy belief and online learning attitudes on PEU and PU of online learning among Chinese vocational college students. Meanwhile, either PEU or PU had a negative effect on practical class satisfaction and a positive impact on theoretical course satisfaction. Besides, by integrating TAM and SOR, along with psychological factors like perceived convenience, curiosity, and self-efficacy, Peng et al. (2024) investigated Chinese university students’ cognition and attitudes towards mobile learning. They found the significant effect of perceived convenience on PEU, PU, attitudes, and intentions to engage with it and the positive influence of curiosity and self-efficacy on usage intentions.

### 2.6 Current research gaps

Much of the literature on AI studies rely on the TAM or the Theory of Planned Behavior (TPB) to account for engagement and usage, but these frameworks can offer a relatively narrow behavioral account when used in isolation (Legris et al., 2003). The flexibility of SOR, however, permits the incorporation of complementary theories to capture richer cognitive and motivational pathways McEown and Oga-Baldwin, 2019. Indeed, scholars emphasize that SOR is adaptable across contexts so long as studies adhere to its core SOR structure (Ngah et al., 2021).

Building on this adaptability, integrating SOR with TAM offers a more complete lens for AI-mediated education. On the cognitive side, PU and PEU can be modeled as organismic appraisals linking information/technology stimuli to adoption and engagement responses. This perspective is especially pertinent when stimuli are defined as chatbot QI (e.g., richness, relevance, accuracy, timeliness, and format), which plausibly influence both cognitive evaluations (PU/PEU) and motivational needs in parallel. Accordingly, the present study positions Doubao’s QI as the stimulus, models PU, PEU, as organismic mediators, and examines engagement—by emotional, behavioral, and cognitive facets—as the response.

Therefore, this study raises the following research questions:

RQ1: To what extent does QI influence learners’ perceptions of Doubao?
RQ2: How do PEU and PU affect different dimensions of student engagement (behavioral, cognitive, and emotional)?
RQ3: What are the mediating roles of PEU and PU in the relationship between QI and multifaceted student engagement?

**Figure 1:**
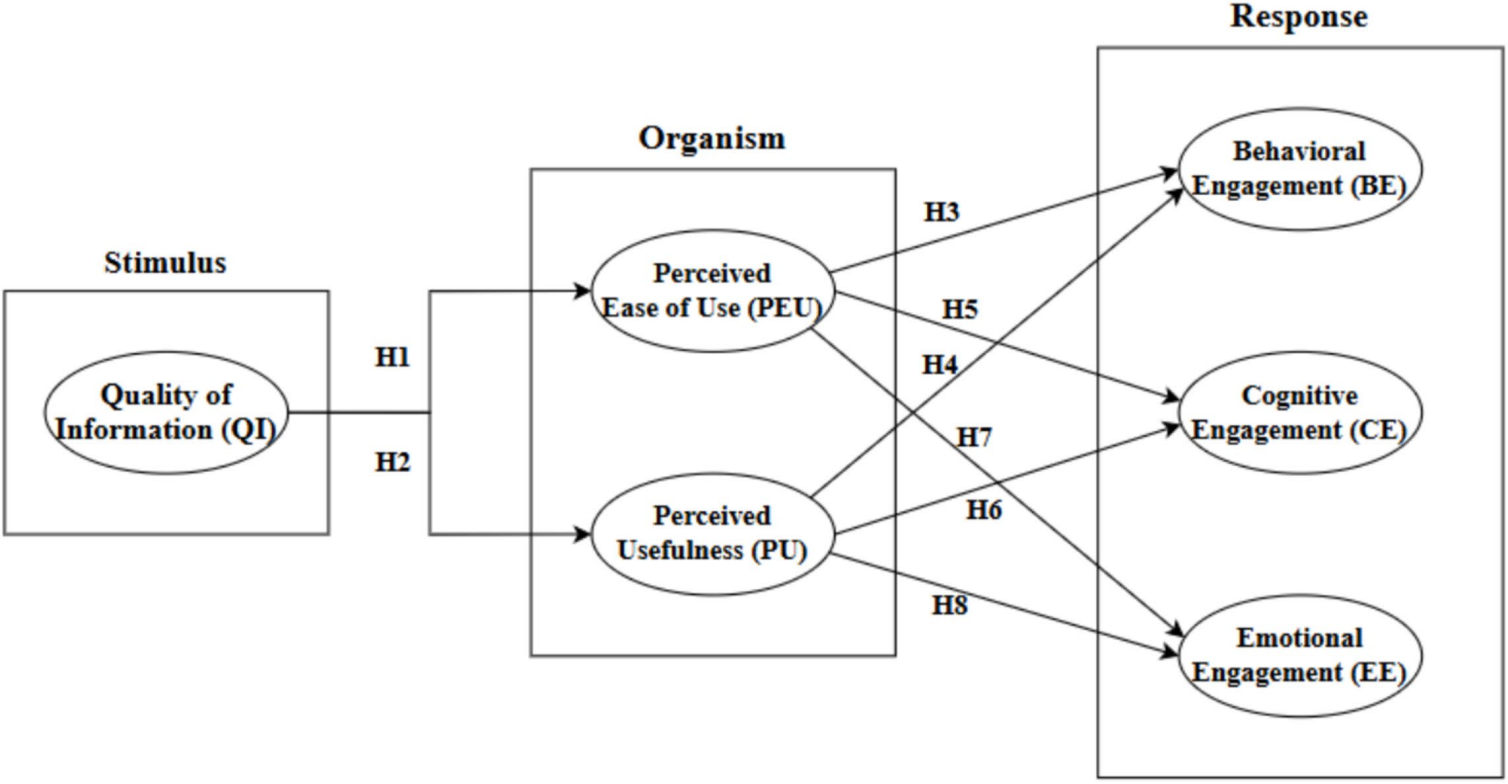
Proposed research model.

Then this study proposes the following research hypotheses (Figure 1):

*H*1: QI has a positive effect on PEU.
*H*2: QI has a positive effect on PU.
*H*3: PEU has a positive effect on Behavioral Engagement (BE).
*H*4: PU has a positive effect on Behavioral Engagement (BE).
*H*5: PEU has a positive effect on Cognitive Engagement (CE).
*H*6: PU has a positive effect on Cognitive Engagement (CE).
*H*7: PEU has a significant positive effect on Emotional Engagement (EE).
*H*8: PU has a significant positive effect on Emotional Engagement (EE).

## 3 Method

### 3.1 Research context and procedure

The study was conducted with the second year English majors at a comprehensive university in Shandong Province, China. To ensure participants had sufficient experiential exposure to the Doubao before measurement, a four-week experiment was implemented. Stage 1: Initial exposure. In the first week, students were introduced to Doubao and its core functionalities. Stage 2: task-based learning: From weeks 2 to 4, students utilized the tool to support collaborative group discussions and linguistic inquiries during class activities. During this period, students were learning the text *The Libido for the Ugly.* Doubao was introduced to facilitate group discussions. Every week, the teacher would give students two questions for group discussions, then they will use Doubao to search for the information. The questions for group discussion are demonstrated at the Appendix. Stage 3: Data Collection: In the fifth week, an online survey was sent to students *via* Wenjuanxing.

Prior to the study, the study was approved by the Ethics Committee of Xihua University. The authors then approached the classes to explain the study’s objectives, emphasizing that participation was entirely voluntary and confidential. Students were assured that they can quit at any time.

### 3.2 Participants and sampling

This study employed a convenience sampling design. Participants were recruited from four intact classes of second-year undergraduate students majoring in English. A total of 140 students were invited to participate. The cohort shared a similar English proficiency level, predominantly at the B2 level of the Common European Framework of Reference for Languages (CEFR). To ensure consistency, all participants were taught by the same instructor from a standardized Integrated English course that utilized the Doubao chatbot for group discussion. This approach minimized potential confounding effects stemming from pedagogical differences and instructor-related bias during the GenAI-assisted group discussions.

The sample consisted of 116 females and 24 males, with a mean age of 20.4 years. The gender imbalance (82.9% female) reflects the typical demographic distribution of English majors in the local national context.

Following the data collection, the research team conducted a rigorous quality control assessment to ensure the authenticity of the dataset. This procedure involved the exclusion of 30 responses that failed to meet our quality benchmarks. Specifically, we filtered out entries with abnormally short completion times (under 60 s), as these typically reflect insufficient engagement or superficial responses. Furthermore, we utilized attention check items and multivariate outlier diagnostics to identify and remove unreliable data (Xie et al., 2025). After this meticulous screening process, 110 valid questionnaires were retained, resulting in a robust effective response rate of 78.6%.

### 3.3 Measurement items

The measurement instruments includes three scales, that is, the QI scale, TAM constructs and engagement scale. To ensure content validity, all items were adapted from established scales and modified to fit the GAI-supported learning context.

QI scale was adapted from Mun and Hwang (2025), measuring five first-order reflective dimensions: accuracy, richness, timeliness, format, and relevance (3 items each). TAM constructs were adapted from Pillai et al. (2024). Student engagement was assessed using the Student Engagement Scale (Reeve and Tseng, 2011), capturing behavioral, affective, and cognitive engagement (12 items).

All items were measured on a five-point Likert scale (1 = “strongly disagree,” 5 = “strongly agree”). To ensure linguistic equivalence, we employed a standard back translation procedure. Two bilingual researchers independently translated the original English items into Chinese, and another researcher back-translated them into English to ensure the original meanings were preserved. The instrument underwent a content validity review by an associate professor in Educational Technology. Subsequently, instead of a large-scale pilot study, we conducted a readability check with five representative participants to identify and correct any ambiguous phrasing, ensuring that all items were clear and comprehensible.

### 3.4 Data analysis

**Figure 2:**
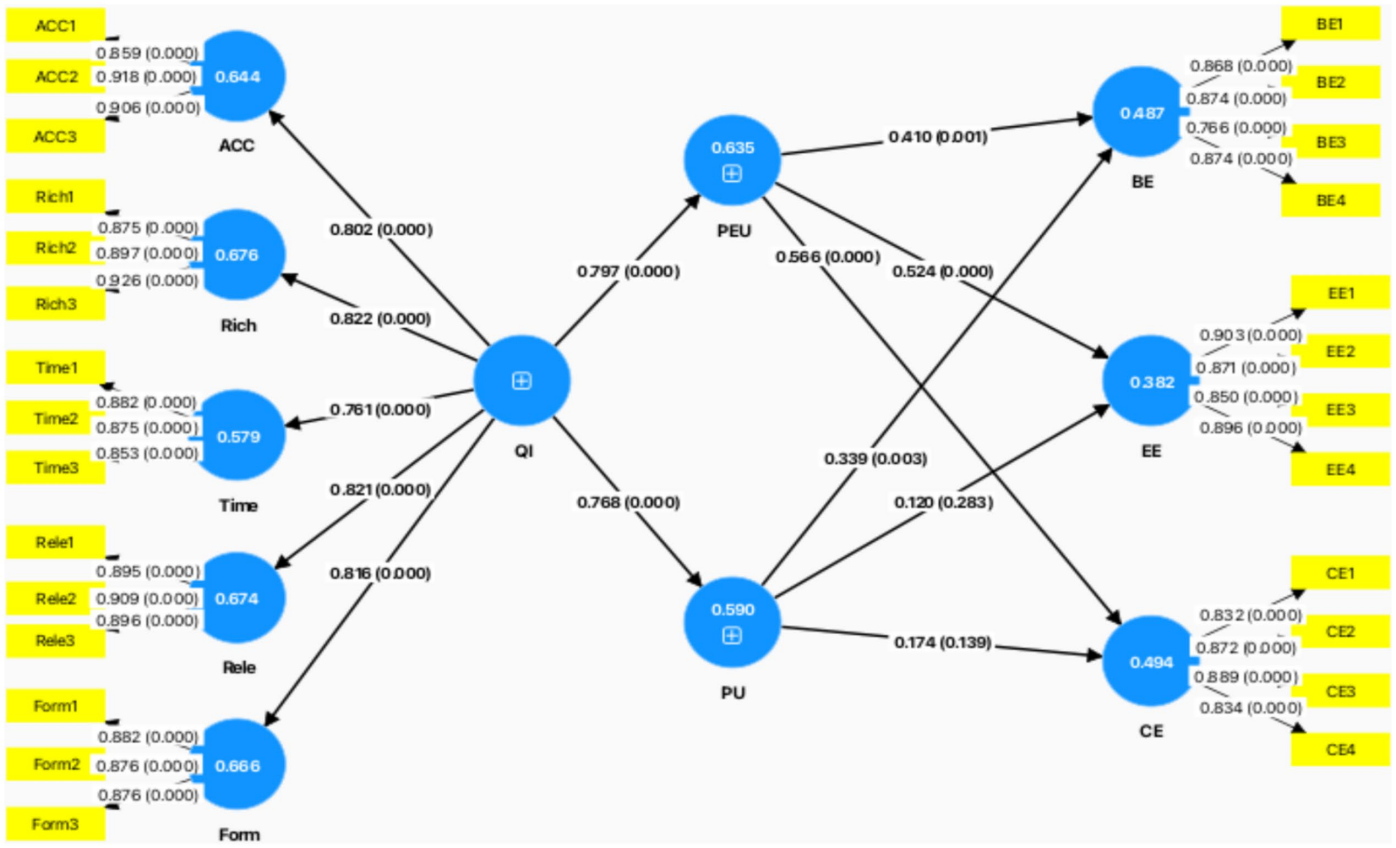
Results of PLS-SEM analysis. (Ringle et al., 2024).

In selecting our research method, we decided to use Partial Least Squares Structural Equation Modeling (PLS-SEM) for two reasons. Firstly, this research contains hierarchical component models and because PLS-SEM can be used to analyse the hierarchical latent variable models (Becker et al., 2012). Secondly, PLS-SEM is also the preferred method when the sample size is relatively small (Hair and Alamer, 2022), which aligns with the limited sample size of students in this study. Since the PLS-SEM algorithm differs from the typical factor-based SEM method, it estimates partial model relationships through iterative OLS regressions, thereby maximizing the explained variance of endogenous latent variables and relaxing the requirements concerning data and relationship specifications (Hair et al., 2021). Therefore, the application of PLS-SEM highlights the model’s predictive power, even with a relatively small sample size. If this study were to be replicated with the same variables, we would expect to observe similar results. Therefore, the study uses SmartPLS v.3.2.6 (Ringle et al., 2024) to test the research models (Figure 2).

## 4 Results

### 4.1 Assessment of measurement model

Four indices were employed to evaluate the outer model: (1) factor loadings, (2) *α* and CR, (3) AVE, and (4) discriminant validity through HTMT of the correlations (Alamer and Marsh, 2022).

Factor loadings, where values higher than 0.70 are generally considered to have good loadings, and those higher than 0.50 are considered acceptable. Cronbach’s α coefficient (α) and Composite Reliability (CR). Here, α is a more conservative reliability indicator, while CR provides a relatively lenient estimate. Average Variance Extracted (AVE), with 0.50 as the threshold for assessing convergent validity. Discriminant validity is assessed using the heterotrait-monotrait ratio (HTMT). When the HTMT value is below 0.90 (ideally below 0.85), it indicates good discriminant validity between constructs.

The factor loadings of the model were all greater than 0.7 (Table 1). As can be seen from Table 2, all Cronbach’s Alpha coefficients exceeded 0.8, indicating good internal consistency among the items within each construct. Composite Reliability (CR) values were all greater than 0.8, further supporting the reliability of the data, and the Average Variance Extracted (AVE) values were all above 0.5, supporting convergent validity. Finally, discriminant validity was verified by checking whether the square root of the AVE for each construct was higher than the correlations between constructs. The HTMT ratios were further tested to ensure they were all below 0.9. Table 2 presents the discriminant validity and HTMT ratio analysis of the study data.

**Table 1:** Descriptive statistics and factor loading.

Items	Mean	Std.	Factor loading	Items	Mean	Std.	Factor loading
ACC1	3	1.181	0.859	PEU5	4	1.135	0.707
ACC2	3	1.251	0.919	PEU6	4	1.266	0.853
ACC3	3	1.194	0.905	PU1	4	1.259	0.859
Rich1	4	1.284	0.868	PU2	4	1.139	0.718
Rich2	4	1.35	0.874	PU3	3	1.139	0.585
Rich3	4	1.262	0.766	PU4	4	1.197	0.778
Time1	4	1.052	0.874	PU5	4	1.151	0.777
Time2	4	1.186	0.832	BE1	4	1.266	0.748
Time3	4	1.129	0.872	BE2	3	1.327	0.784
Rele1	4	1.353	0.889	BE3	3	1.286	0.792
Rele2	4	1.372	0.834	BE4	4	1.296	0.899
Rele3	4	1.294	0.903	EE1	4	1.283	0.907
Form1	4	1.174	0.871	EE2	4	1.346	0.895
Form2	4	1.143	0.85	EE3	4	1.266	0.879
Form3	3	1.341	0.896	EE4	4	1.273	0.894
PEU1	4	1.222	0.882	CE1	4	1.325	0.926
PEU2	4	1.224	0.882	CE2	4	1.255	0.887
PEU3	4	1.241	0.87	CE3	4	1.216	0.87
PEU4	4	1.277	0.822	CE4	4	1.224	0.853

ACC, Accuracy; Rich, Richness; Time, Timeliness; Rele, Relevance; Form, Format; PEU, Perceived ease of use; PU, Perceived usefulness; BE, Behavioral engagement; CE, Cognitive engagement; EE, Emotional engagement. Source(s): The authors’ own work.

**Table 2:** Reliability, validity, and discriminant validity.

Items	Cronbach’s alpha	CR	AVE	The heterotrait-monotrait ratio analysis									
				ACC	BE	CE	EE	Form	PEU	PU	Rele	Rich	Time
ACC	0.875	0.923	0.8										
BE	0.867	0.91	0.717	0.7									
CE	0.879	0.917	0.735	0.684	0.759								
EE	0.903	0.932	0.775	0.621	0.693	0.646							
Form	0.851	0.91	0.77	0.685	0.663	0.665	0.685						
PEU	0.852	0.892	0.583	0.639	0.76	0.794	0.686	0.738					
PU	0.835	0.883	0.602	0.687	0.745	0.682	0.576	0.778	0.872				
Rele	0.883	0.928	0.81	0.635	0.666	0.704	0.796	0.654	0.791	0.745			
Rich	0.883	0.927	0.81	0.616	0.662	0.648	0.688	0.651	0.815	0.746	0.728		
Time	0.84	0.903	0.757	0.618	0.609	0.619	0.601	0.661	0.714	0.642	0.566	0.593	

ACC, Accuracy; Rich, Richness; Time, Timeliness; Rele, Relevance; Form, Format; PEU, Perceived ease of use; PU, Perceived usefulness; BE, Behavioral engagement; CE, Cognitive engagement; EE, Emotional engagement. Source(s): The authors’ own work.

The Variance Inflation Factor (VIF) was calculated using PLS-SEM to address potential multicollinearity issues. According to Hair et al. (2011), VIF values should fall within the range of 0.20 to 5.0. In this study, VIF values ranged from 1.65 to 3.87, indicating that there was no multicollinearity. Subsequently, the quality of the model was assessed to predict the endogenous constructs. This evaluation included several indicators such as the coefficient of determination (*R^2^*), Falk and Miller (1992) suggested that for a model to be considered adequate, the *R^2^* for each latent dependent variable should be at least 0.1. Following the criteria proposed by Hair et al. (2011), *R^2^* of 0.75, 0.50, and 0.25 were utilized as thresholds to describe strong, moderate, and weak levels of explanatory power, respectively. As it is demonstrated in Table 4, the *R^2^* for Rich (0.676), Rele (0.674), and Form (0.666) all approached 0.70, indicating robust explanatory strength. Additionally, ACC (0.644) and PEU (0.635) exhibited moderate-to-high levels of explanatory power. The explanatory power for PU (0.590), Time (0.579), CE (0.494), and BE (0.487) was found to be at a moderate level. Although the explanatory power for EE (0.382) was relatively lower, it remained significantly above the minimum baseline requirement of 0.10. Furthermore, this study reported the Adjusted *R^2^*. Observations revealed that the differences between the original *R^2^* and the Adjusted *R^2^* for all endogenous constructs were negligible (e.g., EE decreased slightly from 0.382 to 0.370). This suggests that the model remains stable after accounting for the number of predictors, indicating the absence of serious overfitting and demonstrating good generalizability.

Another important aspect of model assessment in PLS-SEM is the standardized root mean square residual (SRMR), which helps to prevent model misspecification. SRMR represents the standardized difference between observed and predicted correlations (Kenny, 2020). Although PLS-SEM does not establish a specific threshold for SRMR, it is generally accepted that an SRMR value below 0.10 indicates an acceptable model fit (Kara et al., 2022). In this study, the SRMR was 0.096, indicating a good model fit. RMSEA was 0.088, which was below the recommended threshold of 0.1.

To further evaluate the degree of contribution of specific exogenous variables to their corresponding endogenous constructs, this study calculated the *f^2^* effect sizes. According to the guidelines proposed by Cohen (1988), *f^2^* values of 0.02, 0.15, and 0.35 represent small, medium, and large effects, respectively. It is demonstrated in Table 5, PEU showed a moderate contribution to CE (0.294), EE (0.207), and BE (0.153). In contrast, PU made a small contribution to BE (0.104) and a very minimal contribution to CE (0.028). Notably, the effect size of PU on EE (0.011) fell below the 0.02 threshold, indicating that this specific path provides almost no substantive contribution to the model’s explanatory power.

### 4.2 Evaluation of structural model

Based on the results presented in Figure 2 and Table 3, the hypotheses were evaluated as follows:

**Table 3:** Hypothesis testing.

Hypothesis	Path	Effect	*p* value	Confidence interval 2.5%	Confidence interval 97.5%	Support
H1	QI → PEU	0.797	< 0.001	0.731	0.857	Yes
H2	QI → PU	0.768	< 0.001	0.682	0.848	Yes
H3	PEU → BE	0.41	0.001	0.158	0.636	Yes
H4	PEU → CE	0.566	< 0.001	0.342	0.781	Yes
H5	PEU → EE	0.524	< 0.001	0.298	0.721	Yes
H6	PU → BE	0.339	0.003	0.133	0.574	Yes
H7	PU → CE	0.174	0.139	−0.058	0.404	No
H8	PU → EE	0.12	0.283	−0.084	0.355	No

QI, Quality of information; PEU, Perceived ease of use; PU, Perceived usefulness; BE, Behavioral engagement; CE, Cognitive engagement; EE, Emotional engagement. Standardized path coefficients (β) are reported. *p < 0.05, **p < 0.01, ***p < 0.001. Source(s): The authors’ own work.

**Table 4:** *R*^2^.

Items	*R*^2^	Adjusted *R*^2^
ACC	0.644	0.641
BE	0.487	0.477
CE	0.494	0.484
EE	0.382	0.37
Form	0.666	0.662
PEU	0.635	0.631
PU	0.59	0.586
Rele	0.674	0.671
Rich	0.676	0.673
Time	0.579	0.575

QI has a strong positive effect on PEU (*β* = 0.797, *p* < 0.001), PU (*β* = 0.768, *p* < 0.001). PEU has a significant positive effect on BE (*β* = 0.41, *p* < 0.001), CE (*β* = 0.566, *p* < 0.001), EE (*β* = 0.524, *p* < 0.001). PU has a significant positive effect on BE (*β* = 0.339, *p* = 0.003). However, PU has no significant effect on CE (*β* = 0.174, *p* = 0.139) and EE (*β* = 0.12, *p* = 0.283).

### 4.3 Dominance analysis

**Figure 3:**
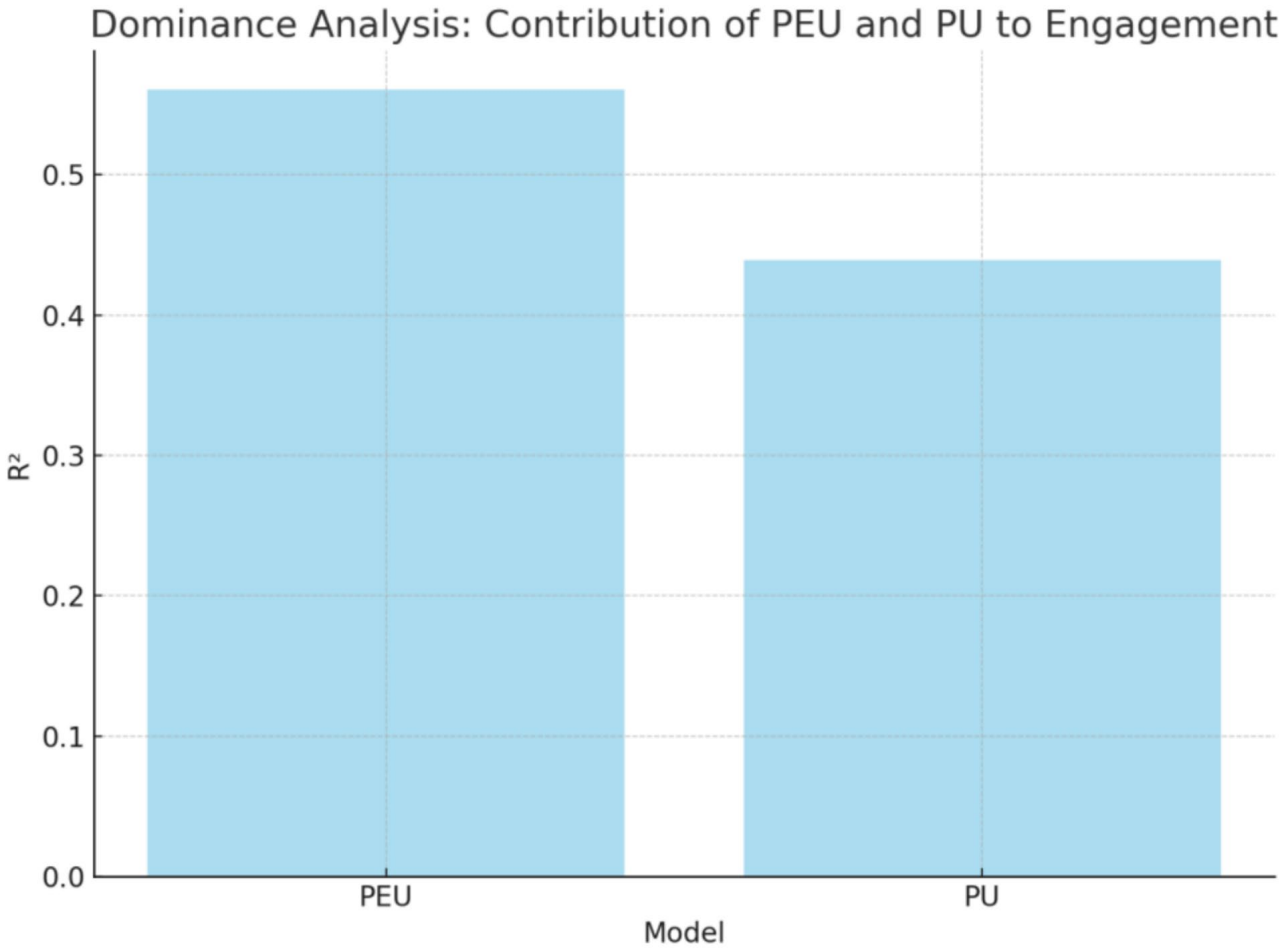
Dominance analysis.

Dominance Analysis (DA) is a statistical method used to evaluate the relative importance of predictor variables in a model, especially when those predictors are correlated. It is a powerful tool for decomposing the total variance explained by a set of predictors and determining how much each predictor contributes uniquely and jointly to the outcome variable (Azen and Budescu, 2003). Unlike traditional regression methods, which may drop variables or provide biased estimates due to multicollinearity, dominance analysis compares the contribution of each variable without removing any predictors, making it more robust in the presence of correlation (Azen and Budescu, 2003). As can be seen from Figure 3, PEU is more important (*R*^2^ = 0.5603) than PU (*R*^2^ = 0.4391) Table 5.

**Table 5:** *F^2^*.

Path	*F^2^*
PEU → BE	0.153
PEU → CE	0.294
PEU → EE	0.207
PU → BE	0.104
PU → CE	0.028
PU → EE	0.011
QI → PEU	1.738
QI → PU	1.441

### 4.4 Mediation analysis

This study further tested the mediating effects of PEU and PU on the relationships between QI, and BE, CE, EE. Based on the results presented in Table 6, the mediation effects were analyzed as follows. QI significantly influences BE through PU (*β* = 0.26, *p* = 0.004). QI significantly influences BE through PEU (*β* = 0.327, *p* = 0.001). However, the mediation effect of QI through PU on CE is not significant (*β* = 0.133, *p* = 0.149). On the other hand, QI significantly influences CE through PEU (*β* = 0.451, *p* < 0.001). For the indirect effect of QI on EE through PU, the results are not significant (*β* = 0.092, *p* = 0.303). Finally, QI significantly influences EE through PEU (*β* = 0.418, *p* < 0.001).

**Table 6:** Results of the mediation analysis.

Path	Effect	*T* value	*P* value	Mediation
QI → PU → BE	0.26	2.889	0.004**	Yes
QI → PEU → BE	0.327	3.3	0.001**	Yes
QI → PU → CE	0.133	1.483	0.149	No
QI → PEU → CE	0.451	5.518	0.000***	Yes
QI → PU → EE	0.092	1.079	0.303	No
QI → PEU → EE	0.418	5.019	0.000***	Yes

QI, Quality of information; PEU, Perceived ease of use; PU, Perceived usefulness; BE, Behavioral engagement; CE, Cognitive engagement; EE, Emotional engagement. Standardized path coefficients (β) are reported. *p < 0.05, **p < 0.01, ***p < 0.001. Source(s): The authors’ own work.

## 5 Discussion

This study aims to explore whether QI will influence BE, CE and EE through PU and PEU. It answers the core question relating to the use of Doubao: To what extent QI (S) drives students’ different psychological states (O) and ultimately impact their engagement(R)? By investigating the influence, this study combines TAM with SOR framework.

The results indicate that QI is a fundamental prerequisite for students’ perceptions and serves as a cornerstone for different dimensions of engagement. The path analysis confirms that QI is a powerful predictor of both PEU (*β* = 0.797, *p* <.001) and PU (*β* = 0.768, *p* <.001), supporting Hypotheses H1 and H2. This finding aligns with the DeLone & McLean (2003) Information Systems Success Model, which identifies QI as a cornerstone of system effectiveness. For students in Shandong’s application-oriented universities, the reliability of GAI content is the primary trigger for their cognitive evaluation of the tool’s ease and utility. High-quality information leads students to perceive GAI tools as more user-friendly, which in turn increases their PEU and PU. Thus, QI serves as a “catalyst” for directly boosting students’ engagement.

The dominance analysis indicates that PEU plays a more important role in engagement, which is consistent with Yu and Liu (2024)’s study. The results demonstrate a significant positive correlation between PEU and BE (*β* = 0.410, *p* < 0.01), CE (*β* = 0.566, *p* < 0.001), and EE (*β* = 0.524, *p* < 0.001). This suggests that when students experience little difficulty in using Doubao, they will become more engaged in learning.

PU contributes to BE (*β* = 0.339, *p* < 0.01). Similarly, the mediation effect of QI-PU-BE is supported. When students use Doubao, they are more likely to engage with the tool if they perceive it as useful. This is consistent with other TAM-based studies, where higher QI predicts stronger PU, thus influencing their engagement (Liu and Ma, 2024; Machdar, 2019). In this study, students used Doubao to help their English learning, therefore PU serves as a direct driving force for BE.

Although PU significantly drives students’ BE, however, there exists no significant relationship with CE (*p* = 0.139) or EE (*p* = 0.283). The unsupported hypotheses reveal a deeper distinction in the types of motivation driving individuals’ engagement at different levels. The most important difference lies in the nature of motivation itself, specifically that PU, as a form of extrinsic motivation, cannot directly translate into an learners’ intrinsic experience. According to Ryan and Deci’s (2002) self-determination theory, human motivation is divided into intrinsic and extrinsic ones. PU, by its very nature, is an extrinsic form of motivation. When students use Doubao to improve their English scores and learning efficiency, this reflects a utilitarian form of motivation, which aligns with Davis (1989) TAM. In contrast, EE and CE are more closely tied to intrinsic motivation, as these forms of engagement often reflect the pleasure and satisfaction individuals experience during the learning process (Fredricks et al., 2004).

A useful tool does not necessarily arouse learners’ interest. Students may use Doubao because it helps them to complete their tasks, therefore, high PU will lead to high BE, but this does not imply that they will enjoy the whole class. Similarly, Doubao may not make them cognitively engaged in the whole class. Students’ PU of a tool is not sufficient to generate the enthusiasm required for deeper engagement. This finding reveals that in the English class, the employment of GAI tools may not help improve students’ emotional engagement and cognitive engagement directly, teachers need to arouse students’ interest by using combing other teaching approaches.

From the perspective of the SOR framework, Doubao serves as an external stimuli that triggers positive cognitive evaluations and behavior responses in learners, which is crucial for promoting English learning (Zhai et al., 2023; Pan et al., 2024). This finding underscores the role of GAI tools not only as functional devices but also as key facilitators of student engagement through psychological mediators.

This study contributes to the literature in two significant ways. Firstly, in the context of modern educational technology, the study extends the TAM into SOR framework, which helps enhance TAM’s explanatory power, particularly in understanding how AI tools influence students’ engagement. Secondly, this study proves that PEU is a central element of students’ experience-driven motivation which plays a crucial role in fostering engagement. The results reveal that students are more likely to engage deeply with AI tools when the tools are easy to use, thus enhancing both their intrinsic motivation and engagement.

## 6 Conclusions and implications

### 6.1 Conclusion

Grounded in the integration of SOR framework and the TAM, this study provides a nuanced understanding of how undergraduates engage with the Doubao in an English learning context. The findings underscore PEU as the most influential determinant of student engagement, exerting consistent and substantial effects across behavioral, cognitive, and emotional dimensions. In contrast, PU functions as a limited, task-oriented driver whose influence remains confined to behavioral engagement, without extending to deeper cognitive involvement or emotional immersion.

These results suggest that in GenAI-supported learning environments, ease of interaction rather than instrumental utility plays a pivotal role in shaping students’ holistic engagement experiences. Merely perceiving an GAI tool as useful is insufficient to foster sustained cognitive effort or emotional attachment; instead, students must experience the technology as intuitive, low-effort, and seamlessly integrated into their learning processes.

### 6.2 Practical implications

From a practical perspective, the findings suggest that educators and developers seeking to promote meaningful engagement with GenAI tools should prioritize usability and interaction simplicity alongside information quality. For students with intermediate or lower language proficiency, intuitive design and low interactional barriers are especially crucial in sustaining motivation and participation. Professional development initiatives should therefore focus not only on introducing AI tools, but also on helping instructors design learning activities that leverage GenAI in ways that minimize cognitive friction and maximize perceived autonomy.

### 6.3 Limitations and future research

This study has several limitations that should be acknowledged. First, its cross-sectional design restricts the examination of changes in students’ engagement over time. Future research adopting longitudinal designs could provide deeper insights into how engagement with AI tools develops through sustained use. Second, the sample size was constrained by the instructional context, as the study involved four intact classes of English majors with a limited cohort size. Although this context-based sampling ensured ecological validity, it may limit the generalizability of the findings. Replication studies with larger and more diverse samples across institutions and disciplines are therefore recommended. The study did not conduct an *a priori* power analysis. This constitutes a methodological limitation as the current sample size might be insufficient to detect smaller effects. Third, the sample exhibited a gender imbalance, with a predominance of female participants, which reflects the demographic characteristics of English education programs (Xie et al., 2025). While this enhances contextual authenticity, caution is warranted when generalizing the results to more gender-balanced or male-dominant populations. Finally, the study relied primarily on self-reported data, which may not fully capture actual engagement behaviors. Future studies incorporating objective usage data or observational measures could offer a more comprehensive understanding of learners’ engagement with AI-supported tools.

## Data Availability

The datasets generated for this study are publicly available in the Open Science Framework (OSF).
