# Supplementary material for: The impact of quality of information on classroom engagement with GenAI tools: integrating TAM and SOR framework
Source: Front Psychol. 2026 Feb 20;17:1756708. doi: 10.3389/fpsyg.2026.1756708 (PMC12963208; doi:10.3389/fpsyg.2026.1756708)
Supplement: Supplementary file 1 [file Data_Sheet_1.docx]

**Supplementary Material**

**Data Sheet 1: Measurement Scales (Appendix A) and Group Discussion Questions (Appendix B)**

Appendix A：Measurement Scales and Items

| **Construct** | **Item Code** | **Measurement Items (Chinese )** | **Measurement Items**  **(English Translation)** | **Sources** |
| --- | --- | --- | --- | --- |
| **Quality of Information (QI)** | | | | **Adopted from Mun & Hwang (2024)** |
| *Accuracy* | ACC1 | 豆包生成的信息是准确的 | The information generated by Doubao is accurate. |  |
|  | ACC2 | 豆包生成的信息是靠谱的 | The information generated by Doubao is reliable. |  |
|  | ACC3 | 豆包生成的信息没有错误 | The information generated by Doubao is error-free. |  |
| *Richness* | Rich1 | 豆包提供的信息是完整的、全面的 | The information provided by Doubao is complete and comprehensive. |  |
|  | Rich2 | 豆包生成的信息具有足够的深度 | The information generated by Doubao has sufficient depth. |  |
|  | Rich3 | 豆包生成的信息内容上非常丰富 | The information generated by Doubao is very rich in content. |  |
| *Timeliness* | Time1 | 豆包生成的信息是最新的 | The information generated by Doubao is current. |  |
|  | Time2 | 我能及时地从豆包获取所需要的信息 | I can obtain the required information from Doubao in a timely manner. |  |
|  | Time3 | 豆包能立马回答我的问题 | Doubao can answer my questions immediately. |  |
| *Relevance* | Rele1 | 豆包生成的信息满足了我的需求，能够切中要点 | The information from Doubao meets my needs and hits the key points. |  |
|  | Rele2 | 豆包生成的信息对我的需求有价值 | The information generated by Doubao is valuable for my needs. |  |
|  | Rele3 | 豆包生成的信息很具体，能直接回应我的问题 | The info from Doubao is specific and directly responds to my questions. |  |
| *Format* | Form1 | 豆包生成的信息排版合理 | The information generated by Doubao is well laid out. |  |
|  | Form2 | 豆包生成的信息呈现形式清晰且易于理解 | The information is presented in a clear and easy-to-understand form. |  |
|  | Form3 | 豆包生成的信息排版简洁明了 | The information generated by Doubao is concise and clear. |  |
| **Technology Acceptance Model (TAM)** | | | | **Adopted from Pillai et al. (2024)** |
| *Perceived Ease of Use* | PEU1 | 豆包灵活且易于使用 | Doubao is flexible and easy to use. |  |
|  | PEU2 | 我在上课回答老师问题的过程中很容易获取和使用豆包 | It is easy to access and use Doubao when answering questions in class. |  |
|  | PEU3 | 我与豆包的互动是清晰且容易理解的 | My interaction with Doubao is clear and easy to understand. |  |
|  | PEU4 | 掌握使用豆包的技能对我来说很容易 | Mastering the skills to use Doubao is easy for me. |  |
|  | PEU5 | 我可以很方便地通过豆包了解英语学习相关的知识和信息 | I can conveniently learn English through Doubao. |  |
|  | PEU6 | 使用豆包完成任务和解决问题会比其他方式更容易 | Using Doubao to complete tasks is easier than using other methods. |  |
| *Perceived Usefulness* | PU1 | 豆包对我的学习有用 | Doubao is useful for my studies. |  |
|  | PU2 | 我觉得借助豆包我能更好地学习 | I feel that I can learn better with the help of Doubao. |  |
|  | PU3 | 豆包会回答我所有的问题，并按照我的期望提供答案 | Doubao answers all my questions and provides answers as expected. |  |
|  | PU4 | 豆包会帮助我提高学习的效率和质量 | Doubao helps me improve the efficiency and quality of my learning. |  |
|  | PU5 | 豆包会为我提供及时的学习问题解决途径，无论我身处何地 | Doubao provides prompt solutions to learning problems regardless of location. |  |
| **Student Engagement** | | | | **Adopted from Student Engagement Scale (Reeve & Tseng, 2011)** |
| *Behavioral Engagement* | BE1 | 我努力在课堂上取得好成绩 | I try hard to do well in this class. |  |
|  | BE2 | 我在课堂上很专心 | I pay attention in this class. |  |
|  | BE3 | 我积极参与课堂的活动 | I participate actively in class activities. |  |
|  | BE4 | 我会认真遵循老师的指令 | I follow the teacher’s instructions carefully. |  |
| *Emotional Engagement* | EE1 | 我喜欢在课堂上学习新知识 | I enjoy learning new things in this class. |  |
|  | EE2 | 在课堂上时，我感觉良好 | I feel good when I am in this class. |  |
|  | EE3 | 这门课我觉得很有趣 | I find this class to be very interesting. |  |
|  | EE4 | 当我在课堂上学到新东西时，我感到很开心 | I feel happy when I learn something new in this class. |  |
| *Cognitive Engagement* | CE1 | 在本门课中，我会尝试把新知识和已有知识联系起来 | I try to connect new knowledge with what I already know in this class. |  |
|  | CE2 | 我会尝试使用不同的策略来理解学习内容 | I try to use different strategies to understand the learning content. |  |
|  | CE3 | 我会尝试把学到的知识应用到实际生活中 | I try to apply what I have learned to real life. |  |
|  | CE4 | 我会自问一些问题来确认自己理解了学习内容 | I ask myself questions to make sure I understand the material. |  |
| Note: *All items were measured on a five-point Likert scale (1 = strongly disagree, 5 = strongly agree).* | | | | |

Appendix B：Group Discussion Questions for GenAI-Assisted Classroom Activities

Week 2:

Who is Henry Louis Mencken? Can you introduce his works?

Do you know about the World’s Top 10 Ugliest Buildings?

Week 3:

Can you show a Pennsylvania State Profile to your partners?

Can you make a comparison of sarcasm, ridicule, irony?

Week 4:

Can you summarize the way of developing a piece of narrative writing?

Should ugliness be preserved as cultural heritage?
